# Scalable medium-density genotyping platforms for cultivar identification, pedigree authentication, marker-assisted and genomic selection, and other applications in strawberry

**DOI:** 10.1093/g3journal/jkag191

**Published:** 2026-07-16

**Authors:** Dominique D A Pincot, Joshua A Sleper, Randi A Famula, Cindy M Lopéz, Dianne Velasco, Maher Al Rwahnih, Allison Krill-Brown, Vance M Whitaker, Steven J Knapp, Mitchell J Feldmann

**Affiliations:** Department of Plant Sciences, University of California, Davis, California, 95616, United States; Horticultural Sciences Department, University of Florida, Wimauma, Florida, 33598, United States; Department of Plant Sciences, University of California, Davis, California, 95616, United States; Department of Plant Sciences, University of California, Davis, California, 95616, United States; Foundation Plant Services, University of California, Davis, California, 95616, United States; Foundation Plant Services, University of California, Davis, California, 95616, United States; Department of Plant Pathology, University of California, Davis, California, 95616, United States; Department of Plant Sciences, University of California, Davis, California, 95616, United States; Horticultural Sciences Department, University of Florida, Wimauma, Florida, 33598, United States; Department of Plant Sciences, University of California, Davis, California, 95616, United States; Department of Plant Sciences, University of California, Davis, California, 95616, United States

**Keywords:** AgriSeq, *Fragaria* × *ananassa*, genotyping, genomic evaluation

## Abstract

A broad spectrum of high-density genotyping approaches, including single-nucleotide polymorphism (SNP) arrays, genotyping-by-sequencing, and whole-genome reduced-representation sequencing, have been shown to perform well in strawberry (*Fragaria* × *ananassa*), despite the inherent complexity of the octoploid genome. While these approaches are effective, their routine deployment in breeding programs can be constrained by cost, computational requirements, and workflow complexity. In parallel, many breeding programs continue to rely on locus-specific assays for marker-assisted selection, resulting in fragmented and inefficient genotyping strategies. Here, we describe medium-density amplicon-based genotyping platforms for strawberry designed to provide cost-effective, turnkey solutions that integrate markers used for marker-assisted selection with genome-wide markers suitable for genomic prediction in a single laboratory assay. These platforms were developed by targeting 1,650 or 4,811 target SNPs via amplicon sequencing, and are interoperable with existing high-density genotyping resources, including a widely used 50K SNP array, thereby facilitating data integration across platforms. We benchmarked their performance relative to the 50K SNP array across breeding-relevant applications, including identity and purity testing, pedigree authentication, marker-assisted selection, and genomic selection, and further evaluated the feasibility of genotype imputation to enhance genome-wide information content. Across analyses, the 1,650- and 4,811-amplicon platforms produced results comparable to higher-density platforms while substantially reducing genotyping cost and analytical overhead. This work demonstrates that targeted amplicon-based genotyping can support efficient, scalable, and integrated genome-informed breeding, enabling the routine application of both marker-assisted and genomic selection within strawberry breeding workflows. Open-source R workflows are provided to support streamlined analyses in breeding contexts.

## Introduction

Genetic markers are the bedrock of modern breeding and genetics (Botstein et al. 1980; Williams et al. 1990; Vignal et al. 2002; Davey et al. 2011). They enable the study of important questions in biology, medicine, and agriculture and the application of predictive breeding approaches in domesticated plants and animals, including cultivated strawberry (*Fragaria × ananassa*), the horticulturally important, allo-octoploid (2n=8x=56) hybrid species targeted in our study. The ability to simultaneously interrogate thousands to millions of genetic markers across an entire genome (Wang et al. 1998; Sebat et al. 2004), in particular, spawned the development of genome-wide association study (GWAS) and genomic prediction methods that have become foundational to the study of qualitative and quantitative traits in natural and experimental populations (Risch and Merikangas 1996; Hirschhorn and Daly 2005; Meuwissen et al. 2016; Crossa et al. 2017). Genomic selection (GS)—an approach where the breeding values of individuals are predicted from genome-wide marker genotypes—has enabled breeders to rethink and redesign breeding schemes to maximize genetic gains by increasing speed, throughput, and operational efficiency (Hayes et al. 2009; Meuwissen et al. 2016; Crossa et al. 2017; Voss-Fels et al. 2019). The scalable application of genomic selection in breeding programs, however, depends on the availability of high-throughput, genome-wide genotyping approaches that are cost-effective and, ideally, that integrate individual genetic markers designed for marker-assisted selection (MAS) in a single laboratory assay.

The genome-wide genotyping approaches available to support purity testing, cultivar identification, pedigree authentication, genomic selection, and other routine breeding and genetic applications in cultivated strawberry rapidly expanded and improved following the sequencing and assembly of the octoploid genome (Edger et al. 2019). The availability of high-quality octoploid genome assemblies enabled experiments to demonstrate that a high percentage of short-read DNA sequences could be unambiguously aligned to subgenome-specific physical addresses in the octoploid genome, and that whole-genome genotyping-by-sequencing was viable (Hardigan et al. 2020). That foundational work informed the development of 50K and 850K Axiom^TM^ SNP arrays using physically anchored probe DNA sequences (Hardigan et al. 2020). Importantly, those studies opened the way for seamlessly applying the full spectrum of experimental and computational methods that are routinely used in breeding and genetic studies of diploid organisms, e.g. genotyping-by-sequencing (GBS), GWAS, *k*-mer GWAS, bulked segregant analysis, identity testing, pedigree authentication, and genomic selection (Michelmore et al. 1991; Risch and Merikangas 1996; Poland and Rife 2012; Crossa et al. 2017; Gezan et al. 2017; Voichek and Weigel 2020; Pincot et al. 2021; Sleper et al. 2025; Vachev et al. 2025).

With turnkey simplicity, a rapid turn around time, accurate allele calling, and minimal missing data, the 50K Axiom^TM^ FanaSNP SNP array has been an important and dependable option for genome-informed studies is strawberry, despite the cost and obsolescence of the technology (Davey et al. 2011; Poland and Rife 2012; Hardigan et al. 2020; Li et al. 2021; https://www.thermofisher.com/order/catalog/product/551041?SID=srch-srp-551041). The cost per sample and design inflexibility, however, has made SNP array genotyping impractical for the scalable implementation of genomic selection in strawberry breeding programs. To overcome those limitations, we developed more cost-effective, but less dense targeted genotyping-by-sequencing (TGS) platforms that interrogate genetic variants in a predefined number of DNA fragments (amplicons) dispersed throughout the genome—amplicon lengths are typically in the 200 to 250 bp range. TGS approaches enable cost-effective sequencing of genetic variants with higher depth and lower missingness than GBS (Poland and Rife 2012), and the incorporation of amplicons that interrogate genetic variants designed for MAS. That flexibility is significant because an increasingly large number of large-effect loci are targeted by MAS in combination with quantitative traits targeted by genomic selection (Gezan et al. 2017; Whitaker et al. 2020; Pincot et al. 2022; Jang et al. 2024; Knapp et al. 2024; Sleper et al. 2025). When properly designed, such platforms offer high data quality, predictable performance, and straightforward laboratory and analytical workflows, making them well suited for high-throughput breeding applications.

Hardigan et al. (2023) developed and tested a 5,000-amplicon platform for strawberry known by the trade name Diversity Arrays Technology^TM^ (DArT) (Kilian et al. 2012; https://www.diversityarrays.com/). Here, we describe TGS platforms, known by the trade name AgriSeq^TM^ (Thermo Fisher Scientific, Waltham, MA, USA; https://www.thermofisher.com/us/en/home.html), that target 1,650 or 4,811 amplicons. While the AgriSeq platforms were developed around the same time as the DArT platform and are of similar density, unlike the DArT platform, they were designed to be a turnkey solution (requiring minimal bioinformatics preprocessing) that was also fully interoperable with the 50K SNP array (Hardigan et al. 2020). Interoperability would allow for easier bidirectional cross-platform use, allowing the wealth of 50K genotypes already published to be leveraged in tandem with AgriSeq genotypes for certain analyses, e.g. training genomic prediction models using legacy data, or imputing newly generated AgriSeq data to allow augmentation of 50K datasets. The AgriSeq platforms’ utility for purity testing, cultivar identification, pedigree authentication, population structure analysis, GWAS, and genomic selection was tested using 50K SNP array genotypes as a benchmark. Their interoperable designs enabled direct, quantitative comparisons of platform performance while isolating the effects of marker density and platform architecture. While marker densities were intentionally below those typically required for GWAS, they captured sufficient genome-wide signal to support accurate prediction of genomic-estimated breeding values (GEBVs). Our benchmarking results show that these breeder-oriented genotyping platforms provide high-quality, reproducible marker genotypes suitable for genomic selection and other routine applications to support the operational needs of strawberry breeding programs. Lastly, well-documented R programing language workflows are provided to support streamlined analyses comparable to those used in our benchmarking studies (R Core Team 2025; https://www.R-project.org/).

## Materials and methods

### Developing the AgriSeq 4.5K platform

We identified potential SNP targets via a platform-agnostic, iterative process. We first created a list of potential target SNPs from the Affymetrix Axiom FanaSNP 50K SNP array (Hardigan et al. 2020), using linkage-disequilibrium statistics and *in silico* position information to guide selection of a panel of approximately 20,000 SNPs in total (“Camarosa” V1 genome; Edger et al. 2019). Linkage disequilibrium (LD) statistics were generated using the genotypic data of 1,580 individuals from the University of California, University of Florida, or USDA ARS GRIN germplasm (the latter set mainly composed of historical *F. × ananassa* heirlooms and *F. chiloensis* and *F. virginiana* ecotypes). We used the R package *SNPRelate* to perform LD-pruning, using the function *snpgdsLDpruning(method = “corr”, maf=0.05, remove.monosnp=TRUE, ld.threshold=0.9)* (Zheng et al. 2012). This resulted in 21,374 SNPs. We assessed the Homozygote Ratio Offset (HomRO) clustering statistic for these SNPs using the Affymetrix Axiom Analysis Suite (v1.1.1.66; Affymetrix, Santa Clara, CA); HomRO measures the degree to which heterozygous and homozygous clusters of a SNP are separated, where a value less than 0 generally indicates poor, overlapping clustering behavior. We excluded any SNP with HomRO less than 0, resulting in a panel of 19,971 SNPs. These steps were repeated, with the final SNP selections re-pooled and manually investigated to ensure that at least 1 marker was present in each nonoverlapping 250,000-basepair (bp) bin across the genome. Twenty-nine SNPs were manually re-added to address gaps in the coverage, bringing the list back up to 20,000 candidates.

These candidates were then sent to a third-party company (LGC Biosearch, Middleton, Wisconsin) for a general quality and performance assessment using proprietary chemistries and software, and the company identified a final panel of 8,316 SNP targets that consistently met their internal testing standards. We then tested the general performance of these SNPs in plant material. We outsourced the genotyping of 95 diverse germplasm accessions (spanning wild ecotypes, old European and American heirlooms, and modern UCD and UFL varieties, 66 of which had been previously genotyped on the 50K SNP array) and 225 seedlings from two bi-parental populations to a third-party company (LGC Biosearch; unpublished data). Using genotypic data from those plants also genotyped on the 50K SNP array, we generated 2,500,000 random SNP subsets, each containing 5,000 SNPs with spacing of 1 SNP every 250,000 bp. We calculated a genomic relationship matrix (GRM) for each set of 5,000 SNPs and estimated the root mean squared error (RMSE) relative to the GRM estimated from the 50K genotypic data. We selected the set of 5,000 SNPs that minimized the RMSE between the GRM computed from the 5,000-SNP and 50K datasets ($RMSE_{\left(5K-50K\right)}=0.027$).

We subsequently submitted these 5,000 target SNPs (henceforth anchor-SNPs) to ThermoFisher Scientific (Santa Clara, CA) for primer design, resulting in the first AgriSeq platform, with amplicons interrogating 4,811 anchor-SNPs; 188 anchor-SNPs were lost during the adaptation to the new chemistry. This platform is publicly listed as “Strawberry 4.5K” in the ThermoFisher Scientific AgriSeq Catalog (https://www.thermofisher.com/us/en/home/life-science/agricultural-biotechnology/agrigenomics/agriseq-targeted-genotyping-sequencing.html, Accessed January 28, 2026).

### Developing the AgriSeq 1.5K platform

We also constructed a second, smaller, and more optimized version of the platform. All estimates, analyses, and filtering were done using custom R scripts. Briefly, we started with 202 individuals from the USDA ARS GRIN, University of California, and University of Florida germplasm collections that had been previously genotyped on the AgriSeq 4.5K platform and 50K SNP array (unpublished data). SNPs with lower missing call rates (<30%) and higher concordance (*ρ*) with prior 50K SNP array genotypes (ρ>0.7) were identified as primary targets (3,252 SNPs in total). We estimated the average minor allele frequency (MAF) of these SNPs using historical 50K SNP array genotypes using genetic databases of accessions from the UF breeding program (n=5,650) and UCD germplasm collection (n=14,010; unpublished data). Using the same algorithm as Sleper et al. (2025), 1,650 equally spaced regions were identified across the genome. Next, a SNP was chosen in each genomic region that had a MAF >0.05 and the highest polymorphism information content (PIC) value across the 202 breeding accessions, calculated per Botstein et al. (1980). Genomic regions (>5% of a chromosome based on *in silico* physical assignments) were identified that did not meet these criteria and were back-filled using SNPs from the 4.5K version when available or a SNP from the FanaSNP 50K (144) or FanaSNP 850K SNP arrays (2) (Hardigan et al. 2020) if no 4.5K version SNP met the criteria. The final designs of the two versions of the new platform overlap by 1,504 anchor-SNPs, with 146 and 3,162 anchor-SNPs unique to the 1.5K and 4.5K versions of the platform, respectively.

We submitted this new set of SNPs to ThermoFisher Scientific (Santa Clara, CA) for manufacturing, resulting in the second publicly-available version of the platform comprised of amplicons targeting 1,650 anchor-SNPs (henceforth, the 1.5K version).

### Plant material & genotyping

For demonstration purposes, we selected four plant populations representing the spectrum of population types a strawberry breeder and researcher may encounter: a breeding population of full-sibling and half-sibling seedlings (henceforth, the Seedling population; n=3,456); a population of clones used as crossing parents (henceforth, the Parental population; n=191); a population of clones generated from tissue culture for nursery propagation (henceforth, the Clonal population; n=96); and a population consisting of diverse *Fragaria* spp. germplasm, including cultivars, heirlooms, and ecotypes (henceforth, the Diversity population; n=190).

To illustrate the application of this new platform to various analyses, we compared and contrasted it with two other technologies: a high-density genotyping solution often used for research and discovery (the FanaSNP 50K SNP array, from which this new platform was derived; Hardigan et al. 2020) and a low-density genotyping solution already used for marker-assisted selection (Kompetitive Allele Specific PCR; KASP).

#### AgriSeq

For the Diversity, Seedling, and Parental populations, we collected leaf tissue for all populations. We lyophilized it at $-70^{\circ }C$ at 100 mTorr for one week using a Benchtop Pro (VirTis SP Scientific, Stone Bridge, NY) before storing it dry at ambient temperature. We then plated at least two 3 mm leaf discs into 96-well PCR plates. For the Clonal population, we collected six leaflets from each mother plant used as a source for meristem excision, just before heat treatment, and dried the samples using Sorbead®orange CHAMELEON®2,050 silica bead desiccant (BASF) at ambient temperature. We plated two 3mm leaf disc punches into a 96-well PCR plate. We then shipped all plates (at ambient temperature) to North American Genomics (Decatur, GA) for DNA extraction, genotyping, and variant calling of the specific anchor-SNP each amplicon was designed to target. The Clonal, Seedling, and Parental populations were genotyped with the 1.5K platform, and the Diversity population with the 4.5K platform. Genotypic information were returned in a simplified allele-dosage format (AA, AB, BB) which we then converted to a 0, 1, 2 format.

We evaluated call rates, genotype counts, and minor allele frequencies prior to any filtering using custom R scripts. Before any other analysis, e.g. purity testing and pedigree authentication, we discarded samples with call rates lower than 50%, resulting in a 1.5K dataset with genotypic information for 2,924 seedlings from the Seedlings population (n=3,269 with technical replicates), 116 parents from the Parental population (n=154 with technical replicates; this includes 23 of 80 possible parents of the Seedling population), and 28 clones from the Clonal population (n=95 with biological replicates). The Clonal and Parental populations overlapped by 13 individuals. Upon filtering the 4.5K dataset, there were 182 accessions from the Diversity population (n=183 with 1 technical replicate).

#### Affymetrix Axiom FanaSNP 50K SNP array

The Diversity population, the Clonal population, and a variation of the Parental population containing more parents of the Seedling population (n = 77 of 80 parents) were genotyped on the Affymetrix Axiom FanaSNP 50K array (Hardigan et al. 2020). DNA was isolated from newly emerging leaves using a previously described protocol (Pincot et al. 2022). Briefly, leaf samples were placed into coin envelopes and freeze-dried in a Benchtop Pro lyophilizer (VirTis SP Scientific, Stone Bridge, NY, USA). Approximately 0.2g of dried leaf tissue per sample was placed into wells of 2.0ml 96-well deep-well plates. Tissue samples were ground using stainless steel beads in a Genogrinder Mini 1,600 (SPEX Sample Prep, Metuchen, NJ, USA). Genomic DNA was extracted from powdered leaf samples using the E-Z 96®Plant DNA Kit (Omega Bio-Tek, Norcross, GA, USA) according to the manufacturer’s instructions. To enhance the DNA quality and yield and reduce polysaccharide carry-through, the protocol was modified by adding Proteinase K to the lysis buffer to a final concentration of 0.2 mg/mL and extending lysis incubation to 45min at $65^{\circ }C$. Once the lysate was separated from the cellular debris, RNA was removed by adding RNase A. The mixture was incubated at room temperature for 5min before a final spin down. To ensure high DNA yields, the sample was incubated at $65^{\circ }C$ for 5min following the addition of elution buffer. DNA quantification was performed using Quantiflor dye (Promega, Madison, WI, USA) on a Synergy HTX (Biotek, Winooski, VT, USA). DNA samples that passed quality and quantity control were genotyped on a GeneTitan HT Microarray System (ThermoFisher Scientific, Santa Clara, CA). SNP genotypes were called using the Affymetrix Axiom Analysis Suite (v1.1.1.66, Affymetrix, Santa Clara, CA), and exported in a simplified numeric allele-dosage format (0, 1, 2). Only samples passing a minimum call rate of 89% were utilized.

#### KASP

KASP-SNP genotyping of the Seedling and Parental populations was outsourced to LGC Biosearch Technologies (Hoddesdon, United Kingdom). These populations were genotyped for two KASP-SNPs: one targeting the FW1 Fusarium wilt resistance locus (FW1−K7; Pincot et al. 2018, 2022) and another targeting the *PF PERPETUAL FLOWERING* locus (PF002; Hardigan et al. 2018). The results obtained from LGC were re-analyzed using a custom R pipeline implementing the Angle of Amplification (*θ*) Method detailed in Brusa et al. (2023), including the recommended discarding of the bottom 20% of datapoints for both HEX and FAM. We re-assigned each individual a KASP-genotype using logistic regression with *MASS:polr(Genotype ∼*θ*)* (v7.3-65; Venables and Ripley 2002).

### Purity testing

Two to eleven replicates (M=2; either technical or biological) of 405 unique individuals were genotyped on the 1.5K version of the platform, totaling 868 samples ($n_{Parental}=83$; $n_{Clonal}=95$; $n_{Seedlings}=690$). We calculated the genotypic concordance of genotypes for all pairwise combinations of individuals using the *caret::confusionMatrix()* function (Kuhn 2008). We estimated the False Discovery Rate ($FDR=\frac{FP}{FP+TP}$) and the True Positive Rate ($TPR=\frac{TP}{TP+FN}$) at different concordance values to determine an optimal threshold delineating concordances belonging to clonal pairs from nonclonal pairs, assuming that all records of sample (and thus clonal) identity were correct. *FP* is the “false positive” count (nonclonal pairs identified as clones), *TP* is the “true positive” count (clonal pairs identified as clones), and *FN* is the “false negative” count (clonal pairs identified as nonclonal). Pairs with concordance values above and below the calculated threshold of 0.86 were assumed to be clonal or nonclonal, respectively.

Eighty of the individuals (n=179 samples in total) were genotyped on both the 1.5K platform and the 50K SNP array. Similarly, 182 unique individuals from the Diversity population were genotyped on both the 4.5K platform and the 50K SNP array; one individual had been genotyped twice on the 4.5K platform (n=183 samples total). For both sets, the 50K genotypic data were subset to those SNPs shared with the corresponding AgriSeq platform, and the analysis was repeated to assess concordance between platforms. We estimated clonal thresholds of 0.77 (1.5K) and 0.79 (4.5K) using the previously described method.

We used the results of Purity testing to remove samples with low concordance with their 50K SNP array analog before subsequent analyses, as these could be due to sampling errors and could introduce noise. We removed 8, 4, and 26 samples from the Clonal, Parental, and Diversity populations.

### Pedigree validation & authentication

We used the parallelized Duo (duo transgression ratios [DTRs]) and Trio (trio transgression ratios [TTRs]) methods detailed in Pincot et al. (2021) to validate and assign parents to individual progeny and families in the Seedling population using two approaches. Briefly, both methods sum the genotypic “transgressions” between parent–offspring duos (the Duo method) or parent–parent–offspring trios (the Trio method); high DTRs or TTRs are unlikely between parent(s) and their offspring. We first used legacy FanaSNP 50K SNP array data for 77 of the 80 possible parents to perform DTR and TTR analyses on 3,269 seedlings from 189 families that had been genotyped on the 1.5K platform. The parental genotypic data were subset to the 1,650 SNPs shared between platforms. We calculated the DTR and TTR for every possible parent-progeny pair and parent–parent–progeny trio, respectively. We repeated the analysis using for 29 of the 80 possible parents that had been genotyped on both platforms. We did not filter the Seedling population for either analysis; seedlings with no parents represented in the Parental dataset were included to assess the analyses’ propensity for false positives (incorrect assignments) or false negatives (nonassignments).

Parent-progeny pairs and parent–parent–progeny trios were classified as “Matching” or “Not Matching” the pedigree record (with the assumption that the pedigree record was correct) (Fig. 3a,b). The *FDR* and *TPR* values were calculated as above, where *FP* was the count of nonparents identified as parents, *TP* was the count of parents identified as parents, and *FN* was the count of parents identified as nonparents. The DTR and TTR values at which *TPR* -*FDR* was maximized were utilized as a threshold for the final parental assignment. Parent-progeny pairs with DTR values or parent–parent–progeny trios with TTR values below the respective thresholds were used to assign parents to progeny. For the first approach, using 50K-derived parental genotypes, we set the DTR threshold to 0.019 and the TTR threshold to 0.039. For the second approach, we used DTR and TTR thresholds of 0.017 and 0.038, respectively, for analyses of 50K-derived Parental data, and 0.011 and 0.030, respectively, for analyses of 1.5K-derived Parental data.

Once individuals had been assigned parents, the assigned parents were tabulated among full-siblings. Parents assigned to fewer than one-third (33%) of all progeny from a family were discarded from the analysis, and the most represented parent(s) were designated as the most probable parent(s) to the family.

### Population structure

As part of validating this new platform, we compared relatedness estimates for those individuals that passed Purity Testing in the Clonal and Parental populations (1.5K platform; n=167 samples representing 76 individuals) and the Diversity population (4.5K platform; n=156 individuals) with those generated when using the higher-density SNP array. For replicated samples in the first dataset, we subset to the sample with the highest call rate. We generated both genomic relatedness matrix (GRM) and imputed genotype matrices for the two platforms’ datasets using the *rrBLUP::A.mat(min.MAF = 0.05, return.imputed=TRUE)* function (v4.6.3; Endelman 2011). Using the GRMs, we estimated the between-platform root mean square error (RMSE) using *Metrics::rmse()* (v0.1.4; Hamner and Frasco 2018) and correlation of diagonal and off-diagonal elements using *stats::cor.test()* (R Core Team 2025).

We further used the Diversity population to perform several additional population-structure analyses commonly used in research: principal components analysis (PCA), admixture analysis, and clustering methods (hierarchical clustering, K-means). These analyses were replicated across genotyping platforms. We first ran PCA on both the previously generated imputed genotypic matrices and GRMs using the *stats::prcomp(center=TRUE)* function (v4.4.3; R Core Team 2025). We assessed the correspondence between PC estimates using *stats::cor.test()* (R Core Team 2025).

For the hierarchical clustering method, we first generated distance matrices of the imputed genotype matrix using *stats::dist(method=“euclidean”)* (R Core Team 2025). We then performed hierarchical clustering on these distance matrices using the *stats::hclust(method=“complete”)* function and visualized the results using the *stats::as.dendrogram()* function (R Core Team 2025). The entanglement of these dendrograms, where an entanglement of 0 and 1 suggests very similar and different dendrogram topologies, respectively, was estimated using the *untangle(method=“step2side”)* and *entanglement()* functions from the *dendextend* R package (v1.19.0; Galili 2015). We utilized the same package for inter-tree comparisons. We calculated the Cophenetic and Baker’s Gamma correlations between the two dendograms using *cor.dendlist(method=c(“baker”, “cophenetic”))*; these correlations range from −1 to 1, with 0 suggesting uncorrelated topologies and a value of −1 or 1 suggesting negatively or positively correlated topologies, respectively. We also estimated the Falkes-Mallow indices by cutting the dendrogram into 2 through 10 clusters and then comparing the cluster assignments of individuals using the *FM_index()* function; for these metrics, a value of 0 and 1 suggest dissimilar and similar dendrogram topologies, respectively.

For the *k*-means clustering method, we first estimated the ideal number of clusters (*k*) using the “elbow” and “silhouette” methods as implemented through the R function *NbClust::fviz-nbclust(FUNcluster = kmeans, method=c(“wss”, “silhouette”))*, using the imputed genotype matrices as inputs (v3.0.1; Charrad et al. 2014). The ideal *k*s were estimated visually to be 2 using both methods; therefore, we generated k-mean clustering assignments using *stats::kmeans(clusters=c(2))* (R Core Team 2025). The concordance of inter-platform cluster assignments was computed using *caret::confusionMatrix()* (v7.0-1; Kuhn 2008).

We performed admixture analysis on the imputed genotypes using the *snmf(alpha=100)* and *Q(K=3)* functions from the *LEA* R package (v3.18.0; Frichot and Francois 2015). For demonstration purposes, we assumed three ancestral populations (k=3) and labeled these according to the nomenclature of Pincot et al. (2021): *F.× ananassa* accessions originating from the University of California breeding program (“California”), *F. × ananassa* accessions originating from other programs (“Cosmopolitan”), and non-*F. × ananassa* ecotypes such as *F. virginiana* and *F. chiloensis* (“Ecotypes”).

### Genome-wide association analysis (GWAS)

We utilized previously reported phenotypic data for Fusarium wilt (FW) resistance and Perpetual Flowering (PF) to run GWAS on three versions of genotypic data for the individuals passing Purity Testing in the Diversity population (n=156): the AgriSeq 4.5K-derived dataset, the FanaSNP 50K-derived dataset, and the 4.5K data imputed to the 50K density using *polyBreedR::impute_L2H()* (v0.47; Endelman et al. 2024). For the imputed dataset, we first utilized *rrBLUP::A.mat(min.MAF=0.05, max.missing=0.80)* to filter and mean-impute missing data. We also removed markers with no *in silico* chromosome or position information as *polyBreedR* relies on position information to guide imputation (J. Endelman, communications; Endelman et al. 2024). After filtering, we used 44,553 SNPs from the 50K dataset for the Diversity population, trained the imputation model, and used it to up-impute 3,458 markers from the 4.5K platform.

For the Fusarium wilt resistance trait, we utilized the resistance scores (1=healthy to 5=dead) reported for 2017 in Online Resource 1 of Pincot et al. (2022); for the Perpetual Flowering trait, we used the flowering habit categories reported in Supplemental File S1 of Brukental et al. (2025) (coded binarily; 0=short-day and 1=day-neutral). In total, 135 accessions had Fusarium wilt resistance data, and 71 had flowering habit data.

We subset the genotypic data to obtain datasets for the two phenotypes. We ran all analyses using these subset datasets as input into *rrBLUP::A.mat(min.MAF=0.05, max.missing=0.80, return.imputed=TRUE)*, the results of which were then fed into the *rrBLUP::GWAS(K=G)* function (Endelman 2011). A p=0.05 false-discovery-rate (FDR) threshold was calculated for each analysis using the *qvalue* R package (as implemented through *rrBLUP*; Storey and Tibshirani 2003). When placed on the -log_10_ scale, the calculated FDR thresholds for Fusarium wilt resistance were: 4.18 (4.5K), 4.19 (imputed), and 4.06 (50K); and for Perpetual Flowering: 3.84 (4.5K), 3.88 (imputed), and 3.86 (50K). The genomic inflation rates for all analyses were less than one, indicating adequate correction of any inherent population structure and related *p*-value inflation ($\lambda_{FW}=0.94-0.99$; $\lambda_{PF}=0.75-0.88$).

### Genome-informed breeding methods

#### Genomic selection

As part of the development process of the 1.5K version of the platform, Sleper et al. (2025) tested many possible sets of 1,650 SNPs in an elite breeding population created at the University of Florida (UFL). We utilized the data in Sleper et al. (2025) to perform GS on a validation population of 20 full-sib families (n=1,460 individuals) using (A) the 1,650 SNPs on the 1.5K version of the new platform (subset from the 50K SNP array), (B) the full complement of 50K SNP array SNPs imputed from the 1,650 marker dataset using *polybreedR::impute_L2H()*, and (C) and the same core set of 10,000 50K SNP array SNPs utilized by Sleper et al. 2025 (J. Sleper, communications; unpublished data; Endelman et al. 2024). We conducted GS with the *rrBLUP::kin.blup(K=G)* function (Sleper et al. 2025).

In addition to re-analyzing the population from Sleper et al. (2025), we also investigated GS in the Diversity population. We leveraged previously published historical data for resistance to *Verticillium dahliae* (Pincot et al. 2020; 2017 estimated-marginal-means in Supporting Material 1) and *Phytophthora cactorum* (Jiménez et al. 2023; cross-year estimated-marginal-means in Supplemental File S1) to assess GS utilizing both platforms. One additional set of analyses was performed utilizing the *P. cactorum* data, in which the *RPC2* locus, which confers partial resistance to the disease, was included as a fixed effect in the prediction model. 107 of the 182 accessions (59%) from the Diversity population had been genotyped on both platforms, had been assessed for both diseases, had been genotyped for the RPC2 partial resistance locus, and had passed Purity Testing; these were the accessions we utilized for these analyses.

For the two diseases, we conducted 107 separate genomic best linear unbiased prediction (G-BLUP) “leave-one-out” cross-validations using the *rrBLUP::kin.blup(K=G)* function in R, where G are the GRMs calculated as previously described (Endelman 2011). For each cross-validation, 106 of the 107 samples was used to train the prediction model, which in turn was used to predict the phenotype of the single remaining sample. Across the 107 cross-validations, all samples were left out of exactly 1 cross-validation. Once all cross-validations were run and single-sample phenotypes predicted and logged, the predictive ability (PA, or correlation between the observed phenotypes and the predicted single-sample phenotypes, where the predicted phenotype is technically a genomic-estimated breeding value) was used to compare the results across platforms.

#### Marker-assisted selection

We utilized the Seedling population, which has been partially genotyped for two KASP-SNPs: one for Fusarium wilt resistance (FW1−K7) and one for Perpetual Flowering (PF002). The FW1−K7 and PF002 KASP-SNPs were designed to target SNPs on chromosomes 2B (AX-184585165; 432,840 bp; Hardigan et al. 2020; Pincot et al. 2022) and 4B (27,044,085bp), respectively. Both KASP-SNPs are correlated with array-SNPs previously identified in association analyses of these traits: AX-184226354 (2B::414,365 bp) for FW1−K7 and AX-184947290 (4B::27,294,311bp) for PF002 (Pincot et al. 2022; Brukental et al. 2025).

We subset the Parental dataset to include only those with KASP-SNP data and that passed Purity Testing, then further reduced the set to include only one replicate per parent, retaining the parent sample with the highest call rate (n=42). The KASP-SNP genotypes for FW1−K7 and PF002 or their corresponding linked array-SNPs: AX-184226354 and AX-184947290, respectively, were then manually added to the Parental dataset used to train the imputation model. We used these training datasets to impute KASP-SNP and Axiom SNP genotypes for the Seedling population using *polybreedR::impute_L2H()* (Endelman et al. 2024). We then evaluated the concordance of imputed KASP- and array-SNPs with the actual KASP-SNP genotypes using *caret::confusionMatrix()* (Kuhn 2008). We created two additional training datasets, the first being composed of 50K genotypic data for the prior 42 individuals. The second was also composed of 50K genotypic data and included both the original 42 individuals and an additional set of 43 randomly selected individuals; however, as not all of the additional individuals had been KASP-genotyped, we only imputed the array-SNP MAS targets.

### Visualizations

All figures were generated using the R packages *dendextend* (v1.19.1; Galili 2015) or *ggplot2* (v4.0.1; Wickham 2016). The visualization capabilities of the latter were further extended with *ggthemes* (v5.1.0; Arnold 2024), *RColorBrewer* (v1.1-3; Neuwirth 2022), *ggchicklet* (v0.6.0; Rudis 2022), and *patchwork* (v1.3.2; Pedersen 2025).

## Results & discussion

### Overview

To guide interpretation of specific results, we organize our evaluation of the AgriSeq 1.5K and 4.5K platforms around core questions and analyses relevant to strawberry breeding and research. Each following subsection begins with a summary of the analysis, the impact on strawberry breeding and research, and the key findings in the first paragraph, before providing the supporting details in following paragraphs, e.g. those pertinent to the specific populations being assessed in this manuscript.

### Genome-wide distribution and coverage of genotyping platforms

**Fig. 1.**
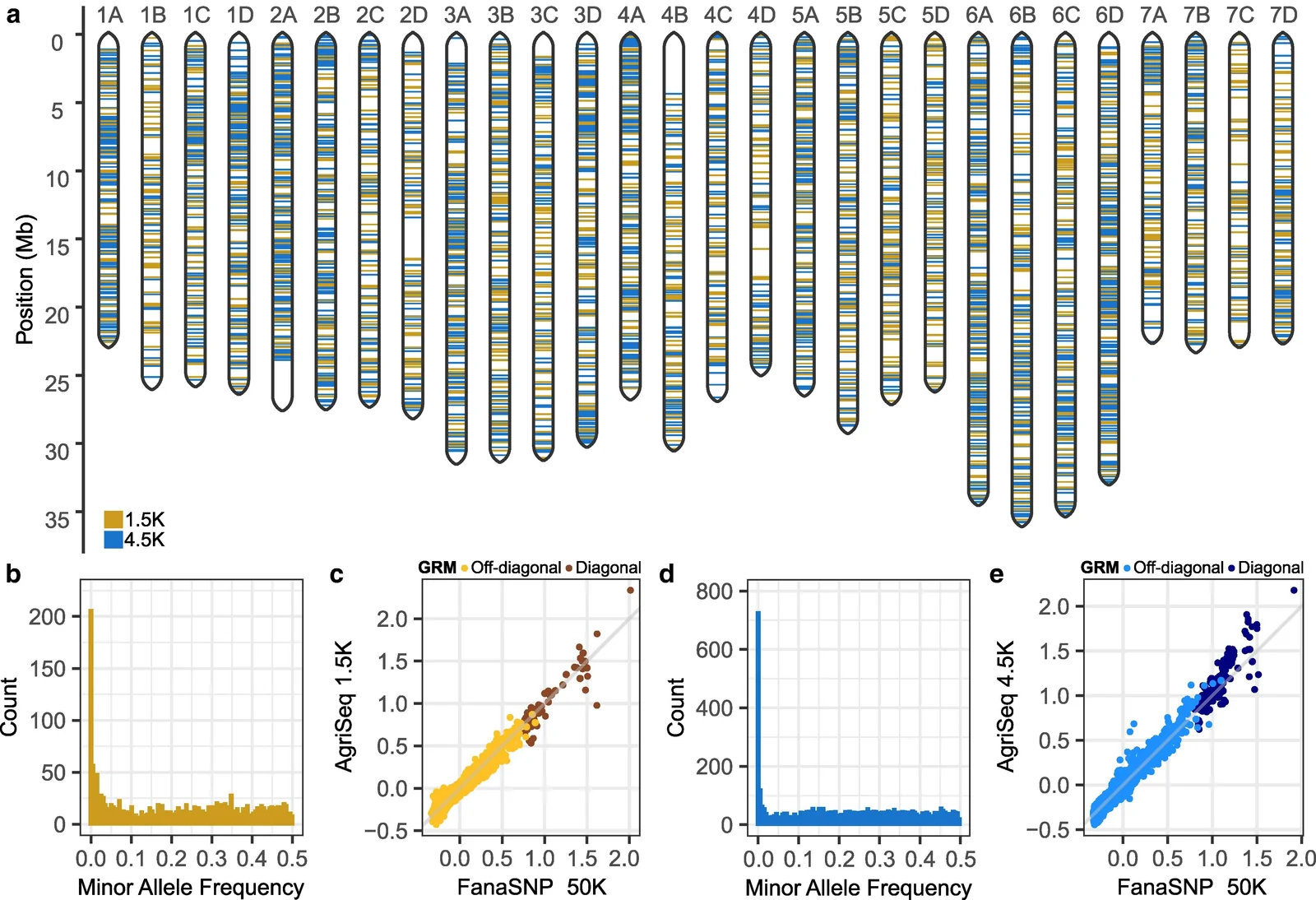
Genomic distribution, allele frequency spectra, and cross-platform concordance of two amplicon-based SNP genotyping platforms (AgriSeq 1.5K and 4.5K) evaluated in cultivated strawberry. a) *In silico* physical positions of SNP-containing amplicons across a *Fragaria × ananassa* reference genome (FaRR1), illustrating genome-wide coverage for both AgriSeq platforms. b) Minor allele frequency (MAF) distribution for SNPs included in the 1.5K marker set estimated from 3,743 individuals. c) Cross-platform comparisons of pairwise genomic relatedness estimates derived from 1.5K platform and 50K SNP array genotypes for 76 individuals. d) MAF distribution for SNPs included in the 4.5K marker set estimated from 190 individuals. e) Cross-platform comparisons of pairwise genomic relatedness estimates derived from 4.5K platform and 50K SNP array genotypes for 156 individuals.

Maximizing genome coverage was a key design choice in our design of these new medium-density platforms as broad and evenly distributed marker representation increases the likelihood of capturing informative proxies for ungenotyped polymorphisms through linkage disequilibrium and co-segregation. This essential feature would increase the ability of this platform to be used for applications such as GWAS, genomic prediction, and imputation to higher marker densities (Habier et al. 2009). To do this, we designed two assays targeting 4,811 and 1,650 polymorphisms distributed across the octoploid genome – the trade names for these are the AgriSeq 4.5K and 1.5K, respectively (Fig. 1a). Both platforms achieved genomewide coverage (>93%) with relatively few large gaps, suggesting adequate density and distribution for downstream genetic and genomic analyses. Detailed descriptions of anchor-SNP selection, subgenome assignments, and chromosome-level marker distributions follow.

Amplicons were anchored by SNPs (anchor-SNPs) selected from a 50K array that were known to be highly informative in diverse populations and distributed across the strawberry genome (Hardigan et al. 2020). Of the 4,811 and 1,650 targeted loci, 4,666 and 1,602 had unambiguous subgenome assignments and physical positions based on sequence alignments and prior genetic mapping results (unpublished data; Supplemental File S4 of Jiménez et al. 2023; Fig. 1a). These loci formed the basis of our estimation of physical genome coverage and identification of gaps.

The 4,811 DNA sequences (amplicons) of the 4.5K platform spanned 0.96Mb (approximately 0.1% of the genome), but the 4,666 amplicons with assigned *in silico* positions were distributed in a manner that covered an estimated 96.6% of the physical length of the genome. The number of markers per chromosome ranged from 74 (7C) to 283 (6D), and the mean inter-locus distance between markers ranged from 120.3kb (1A) to 326.5kb (7C) – the genome-wide mean inter-locus distance was 169.9kb. Most inter-locus differences were shorter than one Mb (n=4,575), though 30 were longer than one Mb, and 5 were longer than two Mb. Two notable gaps were identified: a 3,618.9kb segment on the lower telomere of chromosome 2A and a 4,360.0kb segment on the upper telomere of chromosome 4B that lacked markers.

Using the 1,602 amplicons assigned an *in silico* position, we estimated that the 1.5K platform covers 93.1% of the physical length of the genome. As a consequence of reduced density (4.5K→1.5K), the range of markers per chromosome decreased to between 45 (1A) and 76 (3B), and the genome-wide mean inter-locus distance increased to 479.8kb. There were 10, 111, and 1,518 inter-locus differences longer than two Mb, longer than one Mb, and shorter than one Mb, respectively.

### General performance

Sample and SNP call-rates, allele frequency distributions, and cross-platform relatedness estimates were highly consistent between the AgriSeq 4.5K and 1.5K assays. Both platforms produced relatedness estimates that were strongly correlated with those estimated from the FanaSNP 50K SNP array from which both platforms were originally derived.

Both assays exhibited high sample and SNP call-rates that were highly left-skewed (Table 1). Median sample and SNP call-rates were 0.88 and 0.91 for the 4.5K assay and 0.96 and 0.92 for the 1.5K assay, respectively. Allele frequency distributions were broad for both A and B alleles (ranging from 0.0 to 1.0), with an identical mean minor allele frequency (MAF) of 0.21 for both (Fig. 1b,d). MAF was right-skewed: 3,642 SNPs (76%) of the 4.5K assay and 1,173 SNPs (71%) of the 1.5K assay had a MAF ≥ 0.05 (Fig. 1b,d).

**Table 1.** Summary of populations used for purity (identity) testing, pedigree authentication, population structure, GWAS, MAS, and GS analyses of 1.5K and 4.5K AgriSeq-called SNP genotypes.

Population^a^	Amplicon Number	n^b^	n_F_^c^	n_P_^d^	Purity Testing	Parentage Testing	Population Structure	GWAS	MAS	GS
Clonal	1.5K	96	95	87	✓		✓			
Parental	1.5K	191	154	150	✓	✓	✓		✓	
Seedling	1.5K	3,456	3,269	–		✓			✓	
Diversity	4.5K	190	183	156	✓		✓	✓		✓

^a^ Individuals in the foundation, parent, and diversity populations were clonally propagated. Individuals in the progeny population were seed-propagated.

^b^ Number of individuals genotyped.

^c^ Number of genotyped individuals passing call-rate filters.

^d^ Number of individuals with concordant genotypes.

As part of validating this new platform, we compared the consistency of genomic relatdness estimates for individuals genotyped on both the AgriSeq and 50K platforms, restricting analyses to samples that had also passed Purity Testing (see following section and Table 1; Fig. 1c,e; Hardigan et al. 2023). Relatedness estimates calculated with both platforms were similar to those calculated with the 50K SNP array (r≥0.97; p<0.001; RMSE=0.03−0.06). The strong correlations and minimal differences between relatedness estimates suggest that that the AgriSeq platforms adequately capture genome-wide relatedness patterns and should perform similarly to the 50K array in relevant quantitative genetic analyses, e.g. genomic prediction or population structure analyses, as we later demonstrate.

### Purity testing

Purity testing (“fingerprinting”) is an essential analysis for clonal crops, such as strawberry (Nybom et al. 2014). It can serve multiple purposes: ensuring proper propagation or conservation (Bergelson et al. 2016; Ellis et al. 2018), preventing fraud against growers and consumers (Woutat 1994; Tassie 2010), or enforcing intellectual property (IP) rights or plant variety protection (PVP) (Kumar et al. 2001; Jamali et al. 2019). Purity testing requires a genotyping technology with (1) high reproducibility (or a low genotyping error rate), (2) sufficient marker density to distinguish clonal from nonclonal individuals, and (3) a reasonably low price point. Previously reported fingerprinting panels for cultivated crops typically range from dozens to thousands of markers, depending on the crop’s genome, population dynamics, and breeding system (Padi et al. 2015; Tian et al. 2015; Poets et al. 2020; Yang et al. 2022). The AgriSeq platforms balance these three requirements, and, across all analyses, discriminated clonal from nonclonal individuals with >99.9% accuracy, whether using the AgriSeq platforms alone or by leveraging historical FanaSNP 50K genotypic data (Supplemental Data File 1). They can be used to accurately conduct purity testing in strawberry.

**Fig. 2.**
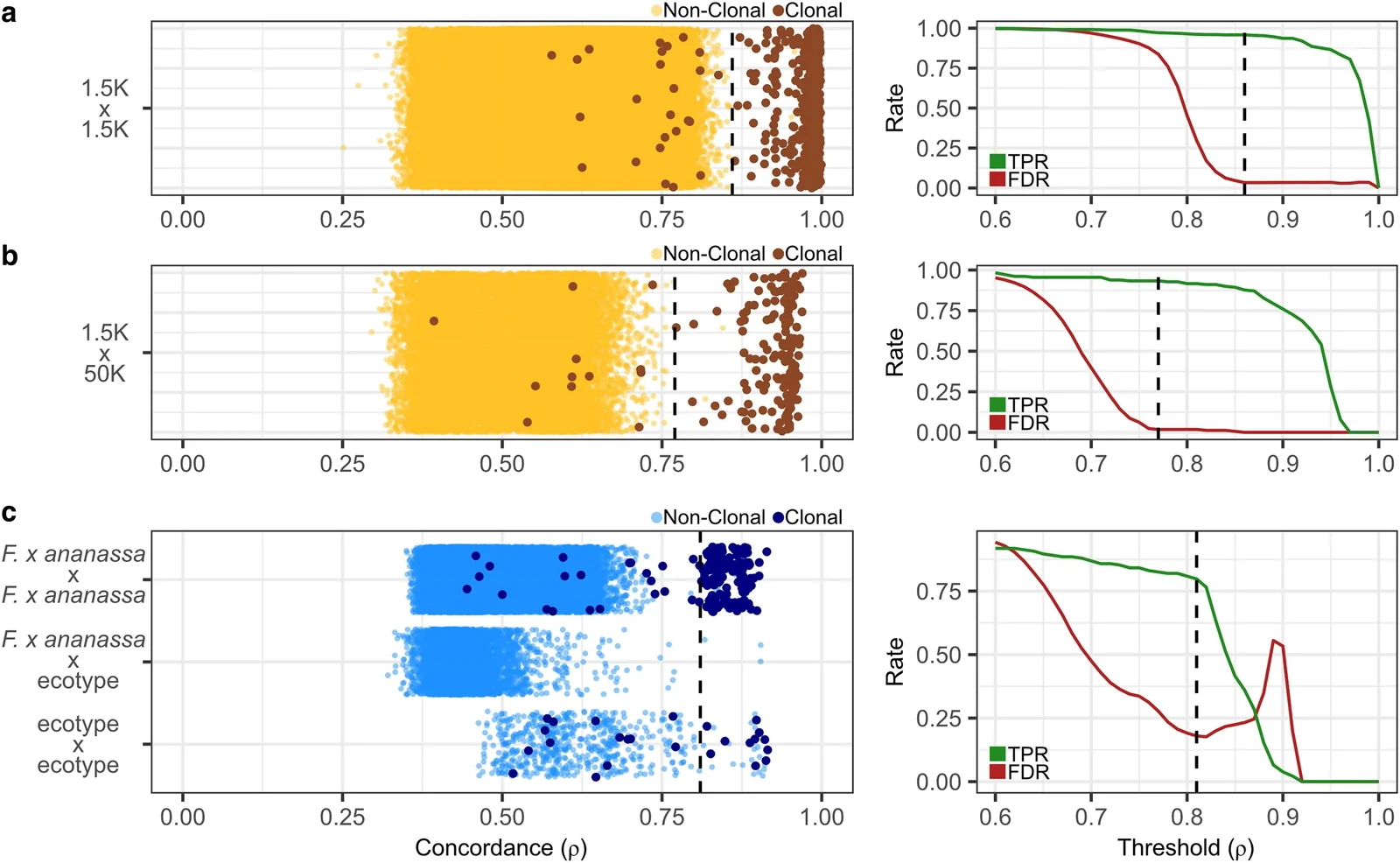
Purity testing of individuals genotyped on the AgriSeq platforms. a) 868 samples of 405 individuals genotyped using the 1.5K platform, b) 179 samples of 80 individuals replicated on both the AgriSeq 1.5K and FanaSNP platforms, or c) 183 samples of 182 individuals replicated on both the 4.5K platform and the 50K SNP array. The rightmost panels show the estimated True Positive (TPR) and False Discovery (FDR) rates at different concordance threshold values, under the assumption that reported clonal identities are correct. The value maximizing the difference between TPR and FDR determined the clonal identity threshold (dashed lines) for each analysis. The rightmost panels show all pairwise concordances between samples in the dataset, with color signifying clonal (darker) or nonclonal (lighter) pairs. The dashed line corresponds to the established clonal identity threshold. Purity testing results can be found in Supplemental Data File 1.

Pairwise concordances (*ρ*) among replicated individuals on the 1.5K platform ranged widely (ρ=0.25−1.00; Mean $\rho =\bar{\rho }=0.62$; Median $\rho =\tilde{\rho }=0.62$; Fig. 2a). Using reported clonal individuals, we estimated the genotyping error rate at 0.03 ($\bar{\rho }=0.97$; $\tilde{\rho }=0.99$). Nonclonal pairs had significantly lower concordances ($\bar{\rho }=0.62$; $\tilde{\rho }=0.62$; t=155.1; p<0.001). Of 596 expected clonal pairs, 571 (95.8%) met or exceeded the threshold for clonal identity, while 375,662 of 375,682 (>99.9%) nonclonal pairs fell below the threshold. These results indicate the 1.5K platform alone provides strong discriminatory power for routine purity testing.

When the 1.5K genotypes were instead compared to legacy 50K SNP array genotypes, clonal and nonclonal individuals again separated clearly (Fig. 2b). Clonal pairs showed higher concordances ($\bar{\rho }=0.91$; $\tilde{\rho }=0.94$) compared to nonclonal pairs ($\bar{\rho }=0.51$; $\tilde{\rho }=0.53$; t=59.5; p<0.001). Although clonal concordances were slightly reduced relative to the within-platform analysis, perhaps reflecting a small penalty when conducting purity testing across platforms, we still identified 167 (93.2%) of 179 clonal pairs (Fig. 2a,b). We flagged the remaining 12 samples (8 Clonal, 4 Parental) for removal in downstream analyses due to low inter-platform concordance and possible sampling issues. 18,255 of 18,258 (>99.9%) nonclonal pairs were correctly identified.

Cross-platform comparisons involving the 4.5K platform produced similar but noisier results (Fig. 2). Clonal pairs and nonclonal pairs were still generally delineated (${\bar{\rho }}_{Clonal}=0.81$; ${\tilde{\rho }}_{Clonal}=0.84$; ${\bar{\rho }}_{Nonclonal}=0.47$; ${\tilde{\rho }}_{Nonclonal}=0.45$; t=46.3; p<0.001; Fig. 2c). Using the established threshold, 146 of 183 (79.8%) clonal pairs and 33,091 of 33,123 (>99.9%) nonclonal pairs were correctly identified.

Upon closer investigation, we found that 22 individuals lacked concordance values exceeding the clonal threshold, though the highest concordance values for half of these (11; 50.0%) was still with their 50K analog. We believed these pairings to still likely to represent actual clonal pairs (and thus did not flag these for removal in subsequent analyses). The others may instead reflect sampling errors, as their highest concordance was not with their 50K SNP array analog. 15 individuals did not have high concordance with their 50K analog, but instead had 26 high concordance pairings with one or more other individuals. 21 (80.7%) of these erroneous pairings involved ecotypes (causing an increase in FDR at ≃0.9 in Fig. 2c), suggesting that there may be reduced discriminatory power in exotic material. To investigate this, we quantified the number of potential discriminatory SNPs (MAF >0.05; missingness <20%) across all ecotypes and subsets of *F. chiloensis* and *F. virginiana*. As a whole, ecotypes had fewer discriminatory SNPs (2,925) than *F. × ananassa* (3,510) (Δ=−585,−17%). Within ecotypes, *F. chiloensis* showed a greater reduction (2,618; Δ=−892,−11%) than *F. virginiana* (2,659; Δ=−266,−9%), consistent with our observations that ∼65% of problematic cases involved *F. chiloensis*. In addition to a lack of discriminative power in certain exotic materials (leading to multiple high concordances), these mismatches may also be due to a combination of sampling error (leading to low concordance with analog).

Finally, 6 samples showed high concordance both with their cross-platform analog and with other samples. We reclassified 2 pairings as clonal based on breeding history: “PI551717” is a colchicine-induced autopolyploid of “PI551716”, and both had high concordance with their corresponding 50K analog and each other’s 50K analog (ρ=0.83−0.84). The highest concordances for “63C120P001” ("Tufts") and “PI552038” (*F. chiloensis* “ILE 02A”) were to their analogs, but they also had high concordance with other samples; those for “PI551436” (“Direktor Paul Wallbaum”) and “PI551953” (“Tribute”) were to other samples, but their concordances with their cross-platform analogs also met the clonal threshold. These cases reinforce that AgriSeq may lack discriminatory power in a specific subset of individuals or genetic backgrounds. In total, we flagged 26 samples, including 14 of 26 ecotypes, for removal in subsequent analyses due to low inter-platform concordance or potential sampling issues.

Overall, the AgriSeq platforms discriminated clonal from nonclonal samples with >99.9% accuracy, whether comparing within the AgriSeq platform or against the 50K SNP array. Although cross-platform comparisons incur a small reduction in clonal concordance, we specifically highlight that interoperability with the 50K SNP array was a key design goal and remains a major advantage, as it enables researchers and breeders to leverage historical (and some publicly available) datasets without the need to re-genotype key material (Pincot et al. 2018, 2020, 2022; Jiménez et al. 2023; Feldmann et al. 2024; Knapp et al. 2024; Brukental et al. 2025).

### Pedigree authentication

Pedigree authentication, or the confirmation of parent–offspring relationships, is another essential analysis in breeding and research. It can be used to create or validate pedigree records, identify pollen or seed contamination, and support IP protection (US District Court for the Northern District of California Case 3:16-cv-02477-VC; Christophi 2017; Pincot et al. 2021). From a bioinformatics perspective, it can also prevent misclassified individuals that could introduce noise and obfuscate causal loci, e.g. in structured mapping populations. As with purity testing, pedigree authentication requires a genotyping platform with high reproducibility, sufficient marker number, and reasonable cost. Across all analyses, the AgriSeq platforms were able to identify parent–offspring or parent–parent–offspring relations and corroborate pedigree records with high accuracy, whether used alone or in combination with 50K array genotypic data (Table 2; Supplemental Data File 1). We are also able to use this analysis to identify systematic issues indicating possible erroneous pedigree records or parental DNA sampling issues in our testing population.

**Table 2.** Pedigree authentication results for the seedling population genotyped on the AgriSeq 1.5K platform, reported at an individual level (n=3,269) and at the familial level (n=189). Parent–offspring duo (DTR) and parent–parent–offspring trio (TTR) analyses were performed on all seedlings using legacy FanaSNP parental data (n=77 of 80 parents) or using a set of parents (n=29 of 80) genotyped on both platforms. Parental assignments can be found in supplemental data file 1.

			# Seedlings Assigned X Parents (Accuracy^a^%)			
Platform^b^	Statistic	Unit	X=0	X=1	X=2	Accuracy^a^ (%)
			Parents Assigned	Parent Assigned	Parents Assigned	
*50K Analysis (n=77 parents)*						
50K	DTR	Individual	11 (31.8%)	490 (66.3%)	2,768 (93.6%)	89.3%
50K	DTR	Family	1 (0%)	35 (61.4%)	153 (96.7%)	89.7%
50K	TTR	Individual	445 (23.7%)	0 (-%)	2,824 (93.3%)	83.8%
50K	TTR	Family	28 (17.9%)	8 (50.0%)	153 (96.7%)	83.1%
*Cross-Platform Analysis (n=29 parents)*						
50K	DTR	Individual	1,196 (96.0%)	1,592 (93.6%)	481 (87.1%)	93.5%
1.5K	DTR	Individual	1,302 (92.5%)	1,494 (91.6%)	473 (86.6%)	91.2%
50K	DTR	Family	67 (97.8%)	95 (95.8%)	27 (94.4%)	96.3%
1.5K	DTR	Family	72 (94.4%)	90 (93.3%)	27 (88.9%)	93.1%
50K	TTR	Individual	2,781 (70.3%)	0 (-%)	488 (88.9%)	73.1%
1.5K	TTR	Individual	2,824 (69.2%)	0 (-%)	445 (87.8%)	71.7%
50K	TTR	Family	163 (69.3%)	1 (100.0%)	25 (96.0%)	73.0%
1.5K	TTR	Family	166 (68.7%)	1 (50.0%)	22 (95.5%)	71.7%

^a^ The accuracy of said assignments (including nonassignments for ungenotyped parents). Accuracy was calculated as the number of “Matching” calls (where an assigned parent met one of the parents listed in the pedigree, including nonassignments for ungenotyped parents) divided by the number of total calls in the analysis. Accuracy was calculated within categories, e.g. 2 Parents Found, or across all categories. This method assumes the pedigree is accurate.

^b^ The genotyping platform from which parental genotypes were obtained: either the AgriSeq 1.5K platform or the FanaSNP 50K SNP array.

Pedigree authentication using legacy parental data from the 50K SNP array worked well across combinations of statistics (DTR: Duo Transgression Ratio, TTR: Trio Transgression Ratio) and units (individuals/families), with overall parental assignment accuracies ranging from 83.1% to 89.7% (Table 2). Most seedlings and families were assigned both parents with high accuracies (93.3% to 96.7%), and most incorrect assignments were nonassignments, e.g. no parents being assigned even though one or both parents are genotyped (Table 2). These incorrect assignments appeared random and nonsystematic, except for seedlings derived from the parents “19C198P037”, “19C168P040”, and “19C135P047”. More than 97% of seedlings derived from “19C198P037” (in pedigree records) were consistently assigned to “19C168P040” and vice versa, suggesting a swap either at the crossing or genotyping stage. Parent “19C135P047” was correctly assigned only 31.5% of the time, with most errors being nonassignments (90.1%). This could signal possible pollen contamination from an un-genotyped parent. These problematic parents were again identified in the family-level analyses: no “19C198P037”- or “19C168P040”-derived families were correctly assigned (but were instead swapped), and only 1 of 8 “19C135P047”-derived families was assigned.

**Fig. 3.**
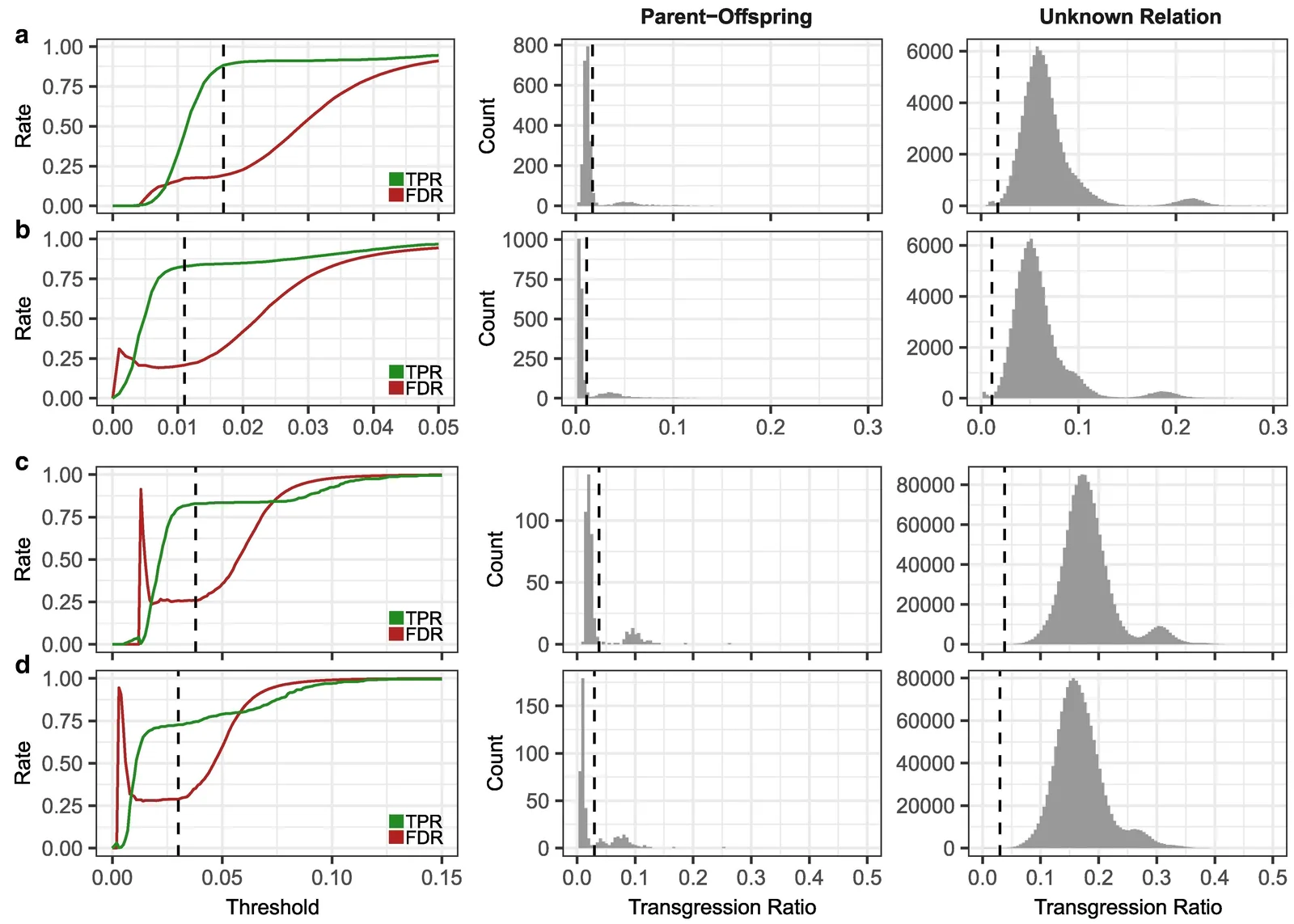
Duo (DTR; Rows a,b) and Trio (TTR; Rows c,d) Transgression Ratio distributions generated between 29 parents genotyped on both the 50K SNP array (Rows a,c) and the AgriSeq 1.5K platform (Rows b,d) platforms, and 3,269 seedlings genotyped using only the latter platform (see Table 2). For each row, the left panel shows the estimated True Positive Rate (TPR) and False Discovery Rates (FDR) across a range of Trangression Ratio values (calculated using known pedigrees and assuming correct pedigrees); the value maximizing the difference TPR and FDR (dashed line) was used as the threshold for seedling parental assignments. The middle and right panels of each row show the Trangression Ratio values for parent-seedling pairs identified and not identified as Parent-Offspring duos (Rows a,b) or Parent-Parent-Offspring trios (Rows c,d) by pedigree, respectively. Parental Assignments can be found in Supplemental Data File 1.

When comparing pedigree authentication results using parents genotyped on both the 1.5K platform and the 50K SNP array, we obtained similar results (Fig. 3; Table 2). DTR and TTR estimates were highly correlated between platforms ($r_{DTR}=0.97$ and $r_{TTR}=0.95$) and assignment accuracies were moderate-to-high (DTR: 91.2%−96.3%; TTR: 71.7%−73.1%; Table 2). Consistent with the fact that fewer parents were genotyped in this comparison, more seedlings were assigned one or zero parents, and the lower-density platform assigned fewer parents overall (∼1%−3%; Table 2). Within each unit and statistic, utilizing the lower-density platform resulted in a mean decrease in accuracy of 2%, driven mainly by an increase in nonassignments (Table 2).

The reduction in genotyped parents affected the TTR statistic more than DTR. As the TTR method assesses two parents jointly, its assignments will be either in pairs (2 parents) or be nonassignments (zero parents). We observed that the TTR analysis generally returned nonassignments when one parent of a parent–parent–offspring trio was not genotyped. Across all combinations of statistics, units, and platforms, the problematic parental samples “19C198P037” and “19C168P040” were again identified because their derived seedlings were assigned to the other parent.

Across both sets of analyses, pedigree authentication using AgriSeq alone yielded slightly fewer overall assignments than when using higher-density data but exhibited comparable accuracy (Table 2). For large-scale authentication, e.g. within a breeding program, the trade-off between slightly fewer assignments and slightly lower accuracy at a lower genotyping cost may be acceptable, especially if multiple individuals from a family can be included in the analysis to enable parallel family-level estimation of parentage.

### Population structure

Population structure analyses have diverse applications in plants, including correcting for inherent population differences in GWAS (Yu et al. 2006; Sul et al. 2018) and establishing the core germplasm sets that become to the focus of research efforts (Choudhury et al. 2014; Liang et al. 2015; Lee et al. 2016). However, technologies targeting limited subsets of SNPs can introduce structural or biological biases, such as uneven genomic coverage or reduced representation of rare alleles (Moragues et al. 2010; Geibel et al. 2021). Because the AgriSeq platforms target SNPs derived from the 50K array, which has already been noted to be biased towards domesticated genetics (Hardigan et al. 2020), we investigated whether these biases were transmitted and exacerbated by the lower marker density. Across multiple population structure analyses, the population structure patterns inferred from AgriSeq data closely matched and correlated with those inferred using the 50K array and with those previously published in papers such as Hardigan et al. (2018, 2021), Hardigan et al. (2021) and Pincot et al. (2021) (Supplemental Data File 2).

Prior genetic investigations and pedigree records suggest that much of the structure across the broader strawberry germplasm corresponds to a combination of two confounded progressions: (1) time, or the known history of domestication and improvement in strawberry, and (2) breeding origin (e.g. improved University of California material versus improved non-California material; Hardigan et al. 2018, 2021; Pincot et al. 2021). Following Pincot et al. 2021, we used the categories “California” (originating from the University of California breeding program), “Cosmopolitan” (non-Californian material, generally conserved public cultivars and heirlooms), and “Ecotypes” (non-*F. × ananassa*) for easier discussion. We further sub-divided the California material into post-1990 versus pre-1990 categories to reflect the impact of known breeding bottlenecks occurring in the late 1980s (Pincot et al. 2021); this pre-1990 material can be considered transitionary (Cosmopolitan becoming early Californian), much like how some of the oldest heirlooms can be regarded as transitionary (ecotypes becoming early Cosmopolitan) (Sjulin and Dale 1987; Pincot et al. 2021). These categories provide an useful framework for subsequent discussion of our results.

**Fig. 4.**
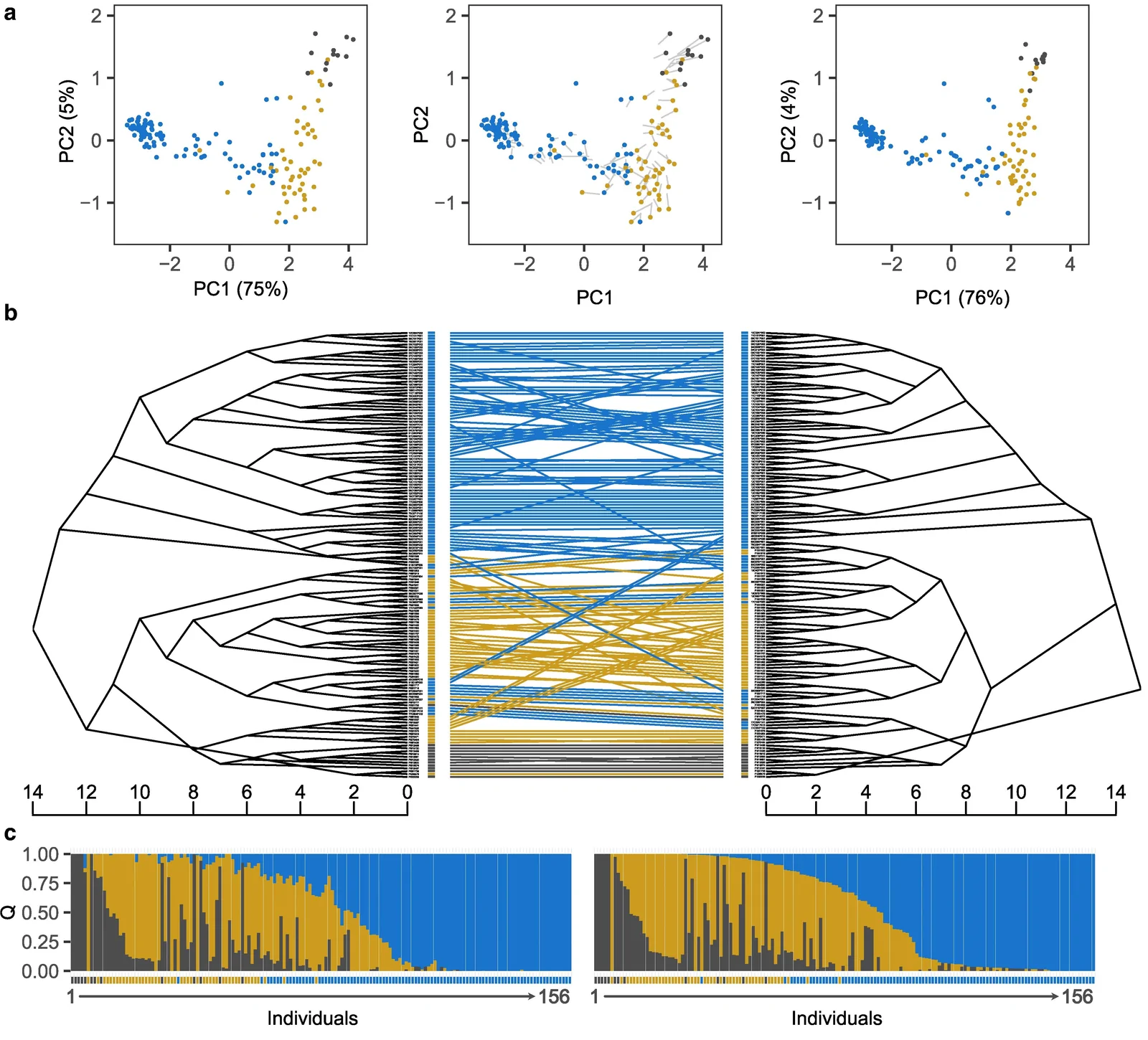
Demonstration of three common population structure analyses of a Diversity population (n=156). Genotypic data were generated using the AgriSeq 4.5K platform (leftmost figures) or the FanaSNP 50K SNP array (rightmost figures). Middle figures a,b) show direct comparisons between the analyses if applicable. Points a), interconnecting lines b), bar chart c), and rugs b,c) are colored following the nomenclature of Pincot et al. (2021): California *F. × ananassa* population (blue), Cosmopolitan *F. × ananassa* population (gold), and other *Fragaria* ecotypes (dark grey). a) PCAs of genomic relationship matrices. The middle plot show the data from both technologies, with 4.5K PC coordinates shown by points, and a light grey line drawn to the corresponding 50K PC coordinates. b) Dendrograms generated using hierarchical clustering. Lines drawn between the two dendrograms indicate the position of individuals within the two dendrograms. c) Admixture analyses showing co-ancestry estimates, assuming 3 ancestral populations (k=3). The admixture categories are colored per their prevalence in individuals belonging to the Pincot et al. (2021) categories. Samples are ordered identically between the two analyses. Population structure results can be found in Supplemental Data File 2.

Principal component analysis (PCA) of the Diversity panel produced a “V”-shaped pattern consistent with previous studies and the domestication and breeding history of the modern strawberry: ecotypes were domesticated into the Cosmopolitan population (forming one leg of the “V”), which was then funneled through selection into the more insular California population (forming the other leg of the “V”; Fig. 4a; Darrow 1966; Hancock 2006; Hardigan et al. 2018, 2021; Pincot et al. 2021). Using genotypic data as input, the first two PCs explained nearly a quarter of the variance for both platforms (PC1=17%; PC2=4%) and were significantly correlated ($r_{PC1}=0.99$; $r_{PC2}=0.99$; p<0.001). We noted that the magnitude of 50K-derived PCs was greater than 4.5K-derived PCs ($\bar{\Delta }PC1=4.3\times$; $\bar{\Delta }PC2=3.9\times$); this is an expected result due to differences in the total estimated variance and eigenvalue scaling that occurs during the PC analysis, driven by the differing number of markers involved in the two datasets (Hastie et al. 2009). Using the GRMs as input, PCs retained the same patterns and correlations ($r_{PC1}=0.99$; $r_{PC2}=0.99$), had perfect correlation with those previously calculated from genotypic data (r=1), and were on a much more similar scale, facilitating direct comparisons ($\bar{\Delta }PC1=1.1\times$; $\bar{\Delta }PC2=1.0\times$; Fig. 4a); however, the variance explained by the first PC increased (4.5K: PC1=75%; PC2=5%; 50K: PC1=76%; PC2=4%).

Hierarchical clustering produced highly similar dendrograms and cluster assignments across platforms (entanglement = 0.05; $r_{Baker}=0.73$; $r_{Cophenetic}=0.80$; Falkes-Mallow indices: ${\bar{y}}_{K=2-10}=0.69$; Range = 0.68−0.84; Fig. 4a). Visual inspection showed that clusters generally distinguished between Californian, Cosmopolitan, and Ecotype individuals. However, some transitional material, e.g. pre-1990 California (grouped with Cosmopolitan), and early Cosmopolitan heirlooms and individuals with recent ecotype ancestry (grouped with ecotypes).

Similarly, K-means clustering (k=2) divided the population into two main clusters: California versus non-California (Cosmopolitan+Ecotype). Concordance of the assigned clusters across the platforms was high (ρ=0.99), and concordance with the California and non-California categories was also high (ρ=0.84−0.85). The majority of discordance involved pre-1990 California individuals (22 of 30 misclassified).

Admixture analysis revealed highly correlated Q-value estimates across platforms for the three designated ancestral categories (r>0.99 and p<0.001 for all; ${\bar{\Delta }}_{4.5K\text{-}50K}=\pm 0.02$ across the three categories; Fig. 4c). We assigned individuals to California, Cosmopolitan, and Ecotype categories based on the highest ancestry proportion, and these assignments were moderately concordant with their category ($\rho_{4.5K}=0.79$; $\rho_{50K}=0.80$). The majority of discordance occurred with transitionary material: the pre-1990 California material (grouping with Cosmopolitan) and the earliest Cosmopolitan heirlooms (grouping with ecotypes).

As previously mentioned, several of these analyses assigned individuals to clusters outside their expected categories. Many of these “misassignments” involve transitional material: the earliest Cosmopolitan heirloom individuals, e.g. PI551681 (“White Carolina”); individuals with recent wild ancestry, e.g. PI616777 (“Sitka”); and pre-1990 California individuals, e.g. “Shasta” and “Wiltguard”. These cases highlight the difficulty of imposing strict categories on a population shaped by gradual transitions and selection: that is, such “failures” to “correctly” cluster these individuals can be better understood as artifacts of human-imposed categories conflicting with gradual population evolution. For example, both early Cosmopolitan and Californian material were developed from a relatively narrow Cosmopolitan base population (Sjulin and Dale 1987; Pincot et al. 2021); it is not surprising that the earliest Californian individuals show a large proportion of “Cosmopolitan” ancestry (${\bar{y}}_{Pre-1950}=66\%-67\%$; ${\bar{y}}_{Pre-1990}=53\%-55\%$) compared to more modern California material (${\bar{y}}_{Post-1990}=7\%-8\%$). This shared ancestry declined over time (at the mean rate of −9% per decade from 1935 to 1995) as selection, inbreeding, and bottlenecks reshaped the insular University of California breeding population into a genetically distinct modern group (Hardigan et al. 2018, 2021; Pincot et al. 2021). Similarly, the earliest heirlooms and those with recent wild ancestry capture the evolution of the wild progenitors of strawberry and the early domesticated population (Ecotype → Cosmopolitan; Sjulin and Dale 1987; Hardigan et al. 2021; Pincot et al. 2021).

### Genome-wide association studies

GWASs are often used to identify marker-trait associations (MTAs) that can be deployed in MAS (Pincot et al. 2018). Because GWAS relies on historical recombination, the density of markers necessary to detect significant MTAs is dependent on genome size, linkage decay patterns, and the distribution of the interrogating markers across the genome (Zhang et al. 2007). Despite the lower-density of the AgriSeq platforms, we were able to successfully replicate prior GWAS results made using the higher-density 50K SNP array, demonstrating their utility for medium-resolution GWAS scans targeting major-effect loci or genomic regions of interest (Supplemental Data File 3). Results could be refined (if needed) via fine-mapping using higher-density genotyping technologies.

**Fig. 5.**
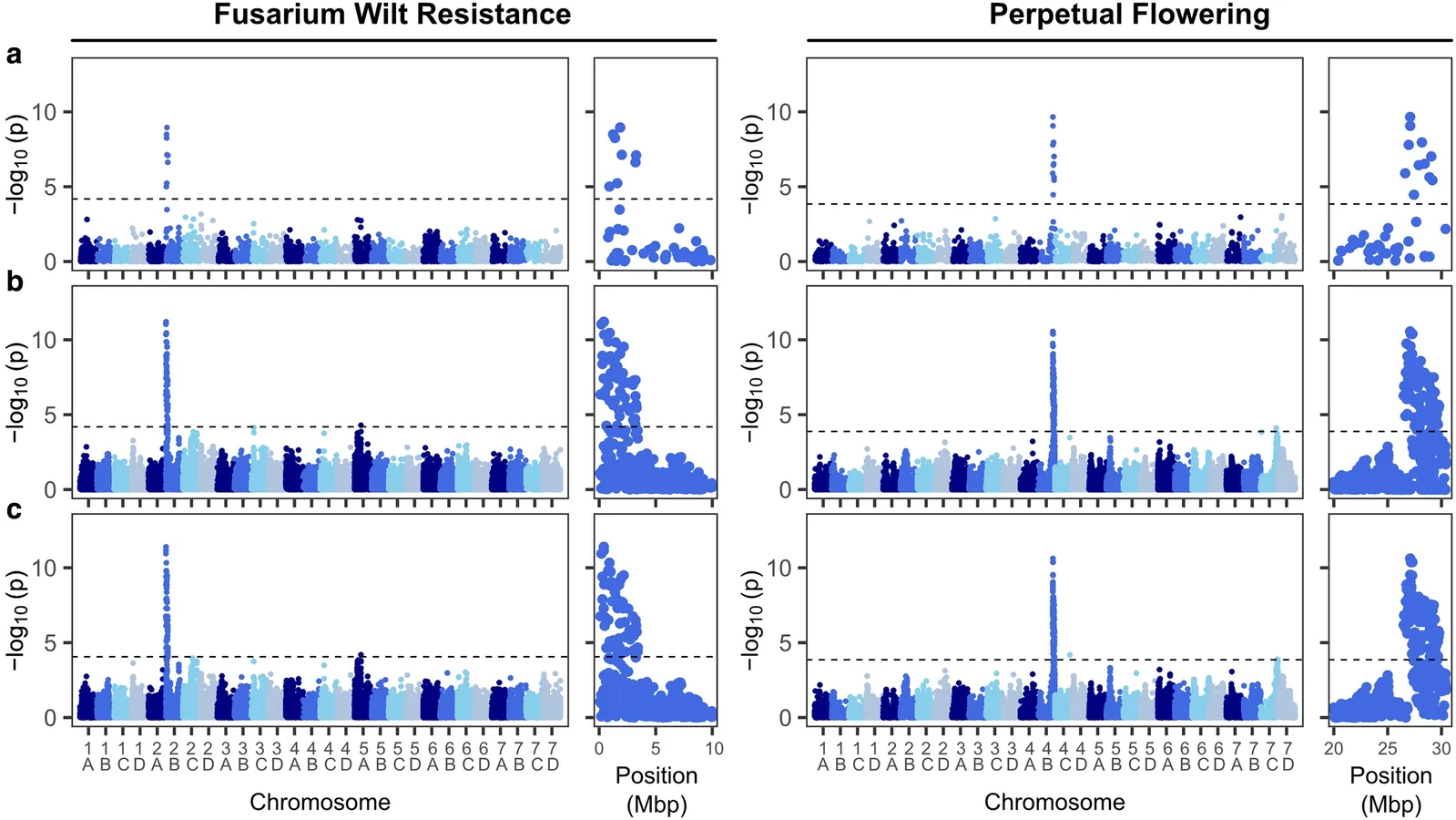
Genome-wide association results for two traits (Fusarium wilt resistance, FW, and Perpetual Flowering, PF) phenotyped in the Diversity population ($n_{FW}=135$; $n_{PF}=71$). Association results were calculated from (Row a) AgriSeq 4.5K genotypic data, (Row b) 4.5K genotypic data imputed to the density of the FanaSNP 50K SNP array, and (Row c) 50K genotypic data. Dashed horizontal lines signify the p=0.05 FDR threshold for each analysis. For each trait × genotyping technology combination, the left (wider) panel shows the genome-wide results and the right (narrower) panel shows a 10-Mb region around the most significant peak (on 2B for FW and on 4B for PF). Association results can be found in Supplemental Data File 3.

Our analyses identified significant MTAs for Fusarium wilt (FW) resistance and the Perpetual Flowering (PF) habit (Fig. 5). These have been previously described (Pincot et al. 2018, 2022; Brukental et al. 2025). For FW resistance, we identified a significant peak on 2B utilizing the 4.5K, 4.5K imputed to 50K, and 50K genotypic datasets. The peak SNPs were AX-184635719 (1,858,179bp) for the 4.5K dataset, and AX-184176344 (413,018bp) for the other datasets, and these SNPs were within 1.4Mb of the AX-184226354, the 50K array-SNP used to track the FW trait (Pincot et al. 2022). For PF, we identified a peak located on 4B that was consistent with prior findings; the peak SNP was AX-184937335 (27,094,218 bp) for all analyses. This peak SNP was located just 0.2Mb from a representative 50K array-SNP (AX-184947290; 27,294,311 bp; Brukental et al. 2025) (Supplemental Data File 3).

For both traits, the genome-wide difference of $-log_{10}(p)$-values for markers conserved between analyses was minimal (${\bar{\Delta }}_{PF}=0.003$; $Median\Delta_{PF}<0.001$; ${\bar{\Delta }}_{FW}=-0.002$; $Median\Delta_{FW}=-0.003$) and highly correlated (FW: $r_{50K\;:\;4.5K\to 50K}=0.97$; $r_{50K\;:\;4.5K}=0.72$; PF: $r_{50K\;:\;4.5K\to 50K}=0.97$; $r_{50K\;:\;4.5K}=0.79$),. These strong correlations reflect that MAF is similar for shared variants and that genotype calls are generally concordant between platforms (Supplemental Data File 3). Similarly, the significant genomic regions identified using the lower-density platform were coincident with those identified by the higher-density platform (Fig. 5; Pincot et al. 2022; Brukental et al. 2025). For FW, the FDR-significant region spanned 908,422-3,269,169bp (4.5K), 108,280-3,312,820bp (4.5K→50K), and 108,280-3,408,245bp (50K). For PF, the FDR-significant regions spanned 26,629,373-29,153,609bp (4.5K) and 26,496,157-29,779,779bp (4.5K→50K, 50K). These overlapping intervals demonstrate the AgriSeq platforms are capable of identifying the same MTA regions as the higher-density array. However, we do caution that while we have presented two examples of successful GWAS, in cases where polygenic architecture with multiple small effect-loci underlies the trait, where causal variants are located in the few regions of the genome not adequately covered by the AgriSeq marker set, e.g. the upper telomere of 4B, or where MTAs are tied to rarer alleles such as those found in nondomesticated germplasm, e.g. ecotypes, GWAS may fail to detect significant MTAs due to the more-limited resolution of the AgriSeq platforms or by its inherent design. In these cases, higher-density genotyping or whole-genome sequencing may be required or preferred.

### Genome-informed breeding

#### Genomic Selection

Genomic prediction and GS is a powerful genomics-informed breeding strategy particularly valuable for traits that are not easily targeted using MAS, such as those lacking known causal variants or with complex genetic architectures. Several strategies have been proposed to maximize the impact of GS in clonal crops like strawberry (Gezan et al. 2017; Yamamoto et al. 2021; Werner et al. 2023), and GS has been validated in experimental strawberry populations for multiple traits (Pincot et al. 2020; Jiménez et al. 2023; Sleper et al. 2025). However, the adoption and routine implementation of GS in breeding programs has been slow (Sleper et al. 2025). A major motivation for developing the AgriSeq platforms was the need for a cost-efficient and user-friendly technology that would enable GS. As part of the development process, we showed that random subsets of 50K SNP array markers at different densities (including subsets of 1,650 markers, equivalent to the number of variants targeted by the 1.5K platform) could enable lower-cost GS in insular breeding populations (Sleper et al. 2025). Briefly, predictive abilities (PA: $r_{y,GEBV}$) and GEBVs were generally comparable and correlated between the AgriSeq 1.5K, 4.5K, and FanaSNP 50K platforms.

We first simulated the prediction performance of the 1,650 (1.5K) SNPs selected for the 1.5K platform by partially replicating the prediction analyses performed in Sleper et al. (2025), using genotypic data from a University of Florida breeding population. Because genotype calls are generally concordant between the two platforms, we believe that subsetting the 50K array to the 1.5K set shared with the AgriSeq 1.5K platform provides a valid proxy for expected performance. Genomic prediction for average fruit weight, the weight culled, and the marketable fruit weight using the 1.5K dataset had a similar PA (${\bar{\Delta }}_{Average}=-0.04$; ${\bar{\Delta }}_{Culls}=-0.01$; ${\bar{\Delta }}_{Marketable}=-0.02$) to prediction done with a denser dataset (10,000 SNPs; Table 3; Supplemental Data File 4). Imputing this 1.5K marker set to a higher density (50K) reduced the difference in PAs (${\bar{\Delta }}_{Average}=-0.02$; ${\bar{\Delta }}_{Culls}=-0.01$; ${\bar{\Delta }}_{Marketable}=-0.02$) (Table 3; Supplemental Data File 4). These results align with prior findings that GS using lower-density marker datasets (with or without imputation) can achieve PAs comparable to those using higher-density datasets (Rogers and Holland 2022; Sleper et al. 2025).

**Table 3.** Predictive abilities ($r_{y,GEBV}$) for fruit weight (grams) in 20 full-sib families (n=1,460 individuals) bred by the university of florida strawberry breeding program (see Sleper et al. 2025 for more information). Predictions were made using the genotypic data of 1,650 SNPs shared between the AgriSeq 1.5K platform and FanaSNP 50K SNP array, genotypic data of those 1,650 SNPs imputed up to the density of the 50K platform (1.5K→50K), and genotypic data for a subset of 10,000 50K SNPs (10K). The results for culls (%) and marketable weight (g) can be found in supplemental data file 4.

			Average Fruit Weight (g)		
Family	n	Season	1.5K	1.5K→50K	10K
17.13	61	2017-18	0.45	0.48	0.50
17.14	83	2017-18	0.40	0.38	0.44
17.17	69	2017-18	0.39	0.41	0.43
17.75	57	2017-18	0.34	0.37	0.32
18.11	85	2018-19	0.46	0.46	0.48
18.57	76	2018-19	0.44	0.45	0.49
18.58	74	2018-19	0.22	0.25	0.32
18.61	85	2018-19	0.50	0.48	0.52
18.72	62	2018-19	0.65	0.60	0.63
19.14	58	2019-20	0.30	0.39	0.39
19.15	57	2019-20	0.31	0.34	0.36
19.17	91	2019-20	0.38	0.41	0.45
19.35	88	2019-20	0.53	0.48	0.58
19.36	86	2019-20	0.29	0.25	0.28
19.47	88	2019-20	0.43	0.47	0.47
20.24	74	2020-21	0.24	0.28	0.27
20.28	77	2020-21	0.40	0.44	0.44
20.32	76	2020-21	0.12	0.08	0.13
20.35	75	2020-21	0.42	0.43	0.45
20.51	38	2020-21	0.17	0.23	0.20

To evaluate performance in a more genetically diverse context than the insular breeding population, we utilized actual 4.5K genotypic data to assess prediction performance in the Diversity population, which has been assessed several times for disease resistance traits and includes modern *F. × ananassa*, heirlooms, and ecotypes (Pincot et al. 2018, 2020; Jiménez et al. 2023; Knapp et al. 2024). We selected a subset of individuals (n=107) that had been phenotyped for resistance against two diseases, Verticillium wilt (VW) and Phytophthora Crown Rot (PhCR), and had also been KASP-genotyped for a crucial partial resistance gene for PhCR (RPC2; Jiménez et al. 2023) (Table 4). Across traits, we achieved similar results using 4.5K or 50K genotypic data. GEBVs were highly correlated ($r_{VW}=0.97$; $r_{PhCR}=0.94$; $r_{PhCR+RPC2}=0.96$), with 4.5K-derived GEBVs within 0.01 of its 50K-derived counterpart on average (Supplemental Data File 4). The general performance of the GS models, as measured by PA ($r_{y,GEBV}$), did not significantly differ across platforms within traits (Table 4; Supplemental Data File 4). For PhCR, PAs (0.15) were lower than previously reported (PA=0.33−0.40; Jiménez et al. 2023) likely due to the much smaller sample size in this study. However, as in prior work, including the RPC2 partial-resistance locus as a fixed effect in the prediction model improved PA to 0.20−0.21. For VW, PAs (0.33−0.34) were comparable to previously published PAs (PA=0.38−0.41; Pincot et al. 2018).

**Table 4.** Predictive abilities ($r_{y,GEBV}$) for Verticillium Wilt (VW) and phytophthora crown rot (PhCR) resistance in the diversity population genotyped with the AgriSeq 4.5K platform and FanaSNP 50K SNP array. For PhCR, prediction models with and without the RPC2 resistance locus included as a fixed effect were tested. Reported PAs are the correlation between observed phenotypes and the phenotypes predicted for 107 individuals using 107 “leave-one-out” cross-validations. Full results can be found in supplemental data file 4.

Population	Trait	4.5K	50K
Diversity	VW	0.34	0.33
Diversity	PhCR	0.15	0.15
Diversity	PhCR + RPC2	0.21	0.20

Together, these analyses demonstrate the AgriSeq platforms can support genomic prediction with performance approaching that of the 50K SNP array across both structured breeding populations and more diverse germplasm. While higher marker densities can provide some gains, the reduce cost of the AgriSeq platforms make them well-suited for routine GS in strawberry breeding programs where thousands or tens of thousands of seedlings could benefit from GS on a yearly basis.

#### Marker-assisted selection

MAS is already routinely implemented in strawberry using low-density platforms such as KASP or high-resolution melting (HRM), targeting loci originally discovered with the higher-density iStraw 35K and FanaSNP 50K SNP arrays (Verma et al. 2016; Pincot et al. 2018, 2020, 2022; Alam et al. 2024; Jang et al. 2024; Knapp et al. 2024; Brukental et al. 2025). Many breeding programs now have both high-density discovery and low-density MAS datasets. We investigated if we could utilize these legacy datasets to impute MAS targets that are not directly captured by AgriSeq in newly-genotyped seedlings. This approach is particularly relevant for programs wishing to implement both MAS and GS simultaneously. Rather than using separate genotyping platforms for MAS and GS, imputation would obviate the need for duplicate genotyping, reduce costs, streamline workflows, and support multiple forms of genetic evaluation. Briefly, in our limited testing, we found that AgriSeq was able to impute MAS targets with moderate accuracy.

To test prediction of MAS targets, we predicted KASP-SNPs and array-SNPs linked to Fusarium wilt (FW) resistance and Perpetual Flowering (PF) traits in 1,605 FW- and 2,567 PF-KASP-genotyped seedlings using imputation (Supplemental Data File 4). We trained the imputation model using 42 individuals from the Parental and Clonal datasets with FW and PF KASP-SNP data. Concordance between predicted and previously obtained KASP genotypes was moderate: 0.66 (FW1 KASP-SNP) and 0.71 (PF002 KASP-SNP). Predicted KASP-SNPs and predicted array-SNPs were highly correlated (FW: $\rho_{KASP\;:\;Array}=0.95$; PF: $\rho_{KASP\;:\;Array}=0.91$), and both produced near-identical concordance results with prior KASP-SNP genotyping ($\rho_{FW}=0.65$; $\rho_{PF}=0.72$). These results suggest that MAS-target predictions using AgriSeq matched real KASP-SNP genotypic data at a moderate accuracy.

Substituting 1.5K-derived for 50K-derived genotypic data in the training set led to predictions that were highly concordant with those obtained using the 1.5K (ρ>0.91 in all cases). These predictions were also concordant with prior KASP-genotyping, and the accuracy was comparable to that obtained when using 1.5K genotypic data alone (ρ±0.03). We also tested the impact of expanding the training set used to train the imputation model by increasing the training set from 42 to 85 individuals (including the original 42). Using the larger training set, concordance between predicted array-SNPs and prior KASP-SNP genotypes improved to 0.74 for FW and 0.76 for PF. These results suggest that the inclusion of more individuals in the training dataset may provide more benefit, e.g. by improving representation of all possible genotypes and genotype combinations, compared to increasing marker density, at least for the tested loci (Howie et al. 2011; Si et al. 2021).

Overall, our results suggest that MAS targets can be predicted via imputation at moderate accuracy using AgriSeq data utilizing a training set augmented with KASP- or array-based MAS target genotypes. While prediction of alternate MAS targets would, of course, need to be validated on a case-by-case basis, these findings demonstrate the potential for AgriSeq-based imputation to unify the MAS and GS strategies under one platform, increasing cost-saving by reducing the need for redundant genotyping, though not without the trade-off of some reduced accuracy.

### Limitations & use-cases

Although the AgriSeq platforms perform well across the various analyses described above, several potential limitations should be acknowledged. As reduced-density assays derived from the 50K array, they inherit (and may amplify) biases present in that platform, which may reduce discriminatory power in certain subsets of germplasm, e.g. ecotypes. While interoperability with the 50K array was a key design goal, cross-platform comparisons still incur some penalty for identity-based analyses such as purity testing and pedigree authentication. The platforms are not suited for fine-mapping, as their marker density limits resolution. Finally, although AgriSeq could enable both routine GS (directly) and MAS (indirectly via imputation), unifying both strategies under a single platform will require appropriate training data and may result in lower MAS accuracy than direct- and targeted-genotyping with low-density technologies like KASP. These limitations reflect expected trade-offs, since these new platforms intentionally reduce marker density to lower costs and enable routine use while also remaining flexible enough to use for several relevant analyses.

In terms of use-case scenarios, the AgriSeq platforms occupy a middle ground: they offer substantially greater analytical power and flexibility than lower-density assays like KASP or HRM while remaining more cost-efficient and scalable than higher-density assays like the 50K SNP array. We have shown that they can detect large-effect marker-trait associations, e.g. via medium-density GWAS scans, and imputation can extend these results to a higher density within closed populations with limited haplotype diversity. This would enhance QTL localization, help identify MAS targets, and guide the development of targeted mapping populations for future fine-mapping with higher-density platforms or whole genome sequencing (Vachev et al. 2025). For more complex traits not conducive to MAS, our results and those of Sleper et al. (2025) suggest that the platform can support GS in both insular (elite) and diverse populations. Programs with existing 50K SNP array data and phenotypic data can “preview” expected AgriSeq GS performance by running GS using the subset of SNPs shared between platforms. For programs wishing to implement both GS and MAS simultaneously, imputation may allow prediction of MAS targets not directly interrogated by the platform with moderate accuracy, reducing the need for additional low-density genotyping; however, we would highly encourage validation and testing on a trait-by-trait basis prior to routine implementation in the breeding cycle.

Higher-density technologies such as the 50K array remain preferable in cases where the goal is high-resolution mapping, e.g. fine mapping, or maximal discriminatory power between individuals, e.g. the construction of crucial core collections or in collections that focus on more exotic germplasm. On the other hand, low-density technologies such as KASP will remain the most cost-effective option for breeding programs focused only on routine MAS without supplemental GS or analysis. In this context, the AgriSeq platforms fill a distinct and valuable niche as an unified, mid-density solution that balances cost, flexibility, and analytical capability.

### Conclusions

We have demonstrated that the newly developed 1.5K and 4.5K AgriSeq platforms can be effectively applied across multiple stages of the strawberry research and breeding pipeline using accessible, open-source analytical tools and software, e.g. R (Supplemental Data Files 5 and 6). The platforms deliver reliable performance in crucial identity-based analyses, providing a practical medium-density solution for confirming clonal identity, e.g. for propagation or conservation, and parentage, e.g. for pedigree validation. Despite their reduced marker density, they also capture the major axes of genetic variation in strawberry, producing clustering patterns, principal components, and ancestry estimates that mirror those obtained from the higher-density 50K SNP array. By balancing accuracy with cost-efficiency and interoperability with legacy 50K datasets, the AgriSeq platforms provide breeders with a practical, reliable, and scalable tool for characterizing relationships, diversity, and population structure, discovering marker-trait associations, and implementing genomic-informed breeding strategies.

## Supplementary Material

jkag191_Supplementary_Data

## Data Availability

The enabling code and data, as well as key results files for the analyses described in this paper, are made available via a ZENODO repository (Pincot et al. 2026; https://doi.org/10.5281/zenodo.18615515), excepting the genotypic data originating from Sleper et al. (2025). The data from Sleper et al. (2025) are protected as intellectual property. As a result, the data associated with that study are not publicly available but are stored in a secure database at the University of Florida. Data can, however, be made available on reasonable request and under a material transfer agreement. Please see the Supplementary Data Access Procedure file for the appropriate procedure. The remaining supplements are briefly described here; a more detailed manifest is available in the ZENODO repository, and each supplemental Excel (.xlsx) file contains a worksheet (labeled “Legend”) that describes the contents of all other worksheets. Supplemental Data File 1: An Excel (.xlsx) file containing the results for identity-based analyses (purity testing and pedigree authentication). Supplemental Data File 2: An Excel (.xlsx) file containing the results for population structure analyses, e.g. GRM, PCA, k-means, STRUCTURE. Supplemental Data File 3: An Excel (.xlsx) file containing the results for genome-wide association analyses. Supplemental Data File 4: An Excel (.xlsx) file containing the results for breeding methodology analyses, e.g. genomic selection and prediction of marker-assisted selection targets via imputation. Supplemental Data File 5: A .zip file containing all R code utilized for the analyses in this this paper. All enabling inputs (including AgriSeq 1.5K, AgriSeq 4.5K, and FanaSNP 50K genotypic matrices) are provided. Supplemental Data File 6: A .zip file containing a much simplified R script detailing core analyses performed in this paper. Introductory and computationally inexpensive example datasets are provided. Supplemental material available at G3 online or at the previously described Zenodo database.
