# Supplementary material for: Scalable medium-density genotyping platforms for cultivar identification, pedigree authentication, marker-assisted and genomic selection, and other applications in strawberry
Source: G3 (Bethesda). 2026 Jul 16;16(9):jkag191. doi: 10.1093/g3journal/jkag191 (PMC13535441; doi:10.1093/g3journal/jkag191)
Supplement: Supplementary file 1 [file jkag191_supplementary_data.pdf]

<u>Supplementary Data Access Procedure:</u>

Requesting Access

Requests for data access should be submitted by e-mail to the corresponding author of Sleper et al. (2025) (https://doi.org/10.1002/tpg2.20550). Requests may only pertain to the portion of data re-analyzed for Pincot et al. (2026). Requests must include:

- Names and institutional affiliations of all individuals who will access the data.
- A detailed description of the proposed research and how the data will be utilized, including methods of analysis.
- Information on how results will be used, including whether the work may lead to publications or intellectual property.
- A description of how confidentiality and chain-of-custody will be maintained.

Parties Responsible for Reviewing Requests

Requests will be evaluated by the corresponding author of Sleper et al. (2026) and director of the University of Florida's Institute of Food and Agricultural Sciences (UF/IFAS) Strawberry Breeding program (V. Whitaker), the data scientist who authored Sleper et al. (2026) (J. Sleper), and any relevant UF/IFAS staff who may be impacted by the data-sharing, as determined by V. Whitaker.

Evaluation Criteria and Process

The corresponding author will forward each request to the reviewing parties. Requests will be evaluated based on:

- Overlap with ongoing research in the UF/IFAS strawberry breeding program.
- Potential impacts on or conflicts with the UF/IFAS strawberry breeding program's commercial interests.

Reviewers may request clarification before issuing a recommendation. If consensus cannot be reached, the final decision rests with V. Whitaker. A written approval or denial, including reasons for denial, will be provided within 60 days.

Conditions for Data Access

- Execution of a material transfer agreement (MTA) provided by the granting parties, including written assurance of confidentiality and acceptance of all associated liabilities.
- Submission of all research results and any public-facing materials to the corresponding author for review at least 60 days prior to publication; reasonable

revisions requested by the corresponding author must be accommodated, including the de-identification of any UFL/IFAS material.

- No use or licensing of the research results for commercial purposes.
- Intellectual property arising from the research will be jointly held by the requesting party and members of the original research project.
- The requesting party may not enter into any data-sharing agreements without written approval from the granting parties.

Reasons for Denial

Access may be denied if:

- The proposed research either overlaps with ongoing work in the UF/IFAS strawberry breeding program or conflicts with the commercial interests of the UF/IFAS strawberry breeding program.
- The research outputs will be used, patented, or licensed for commercial or university interests by the requesting party or its affiliates.
- The requesting party cannot guarantee confidentiality of the data.
